# Reparative Dentinogenesis Induced by Mineral Trioxide Aggregate: A Review from the Biological and Physicochemical Points of View

**DOI:** 10.1155/2009/464280

**Published:** 2009-12-28

**Authors:** Takashi Okiji, Kunihiko Yoshiba

**Affiliations:** Division of Cariology, Operative Dentistry & Endodontics, Department of Oral Health Science, Niigata University Graduate School of Medical & Dental Sciences, 5274 Gakkocho-dori 2-bancho, Chuo-ku, Niigata 951-8514, Japan

## Abstract

This paper aims to review the biological and physicochemical properties of mineral trioxide aggregate (MTA) with respect to its ability to induce reparative dentinogenesis, which involves complex cellular and molecular events leading to hard-tissue repair by newly differentiated odontoblast-like cells. Compared with that of calcium hydroxide-based materials, MTA is more efficient at inducing reparative dentinogenesis in vivo. The available literature suggests that the action of MTA is attributable to the natural wound healing process of exposed pulps, although MTA can stimulate hard-tissue-forming cells to induce matrix formation and mineralization in vitro. Physicochemical analyses have revealed that MTA not only acts as a “calcium hydroxide-releasing” material, but also interacts with phosphate-containing fluids to form apatite precipitates. MTA also shows better sealing ability and structural stability, but less potent antimicrobial activity compared with that of calcium hydroxide. The clinical outcome of direct pulp capping and pulpotomy with MTA appears quite favorable, although the number of controled prospective studies is still limited. Attempts are being conducted to improve the properties of MTA by the addition of setting accelerators and the development of new calcium silicate-based materials.

## 1. Introduction

The aim of direct pulp capping is to maintain the vitality and function of the dental pulp following its exposure to the external environment. Although calcium hydroxide-based materials have been extensively used for this procedure because of their potential to induce hard-tissue repair and subsequent dentin bridge formation [1], mineral trioxide aggregate (MTA) has recently received much attention as a good substitute for calcium hydroxide-based materials and has demonstrated promising clinical outcomes (reviewed in [2]). 

MTA is a bioactive material that was developed in the early 1990s, originally as a retrograde filling material, and first appeared in the dental scientific literature in 1993 [3]. Since then, the indications for MTA have expanded significantly. Currently, MTA is used to seal off exposed pulps and various communications between the root canal system and surrounding tissues for a variety of indications such as root-end filling, perforation repair, and apexification [4]. MTA is a modified preparation of Portland cement [5–8], which is the basic ingredient of concrete and mortar that may never have been used as a dental material before the development of MTA. Currently, two different preparations of MTA are available: the original preparation is grey-colored (GMTA); whereas, a white preparation (WMTA) was recently introduced to address esthetic concerns. 

A large number of studies have disclosed that MTA shows favorable biocompatibility and has physical properties suitable for dental application such as good sealing ability. However, the basic question of why such materials induce the hard-tissue repair of exposed pulps has not yet fully been answered. Thus, the purpose of this paper is to summarize the biological process of pulp tissue repair and then review the available literature regarding the ability of MTA to induce reparative dentinogenesis from both the biological and physicochemical points of view.

## 2. Pulp Wound Healing and Tissue Repair

**Figure 1:**
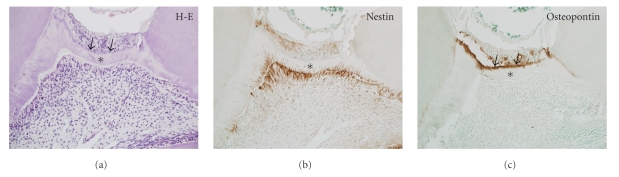
Dentin bridge formation in rat molar at 14 days after direct pulp capping with MTA: H-E staining (a), immunohistochemistry of nestin (b), and osteopontin (c). (a) A thin layer of fibrous matrix (arrows) is followed by a dentin-like matrix (∗) with tubular structures pulpally lined with odontoblast-like cells. (b) The odontoblast-like cells intensely express nestin, an intermediate filament expressed in differentiated odontoblasts. Their processes also show immunoreactivity for nestin in the tubular matrix (∗). (c) Osteopontin immunoreactivity is detected in the superficial fibrous matrix (arrows), but not in tubular dentin-like matrix (∗).

Dental pulp possesses a natural tissue repair potential, which leads to the formation of reparative dentin. It has been well documented that dental pulp possesses the ability to form a hard-tissue barrier (dentin bridge) after direct pulp capping or pulpotomy. During reparative dentinogenesis, the original odontoblasts at the exposure site are destroyed and replaced by newly differentiated odontoblast-like cells [1, 9–11]. Pulpal wound healing involves stem/progenitor cells migration to the injured site and their subsequent proliferation and differentiation into odontoblast-like cells. Reparative dentinogenesis is often initiated by the formation of a fibrodentin matrix, which is atubular and/or irregular and is associated with cuboidal cells. The formation of a tubular dentin-like matrix by elongated and polarized odontoblast-like cells takes place later (Figure 1).

Despite extensive studies, the molecular signaling involved in cell differentiation during reparative dentinogenesis has still not been fully characterized. During tooth development, odontoblast differentiation is controled by specific basement membrane-mediated epithelial-mesenchymal interactions [12–14]. Fibronectin, an extracellular matrix glycoprotein found in association with the dental basement membrane, appears to play a crucial role in the terminal differentiation of odontoblasts [15, 16]. On the other hand, during reparative dentinogenesis when the basement membrane or dental epithelium is absent, the adhesion of progenitor cells to an appropriate surface (scaffold) may be a critical requirement for the differentiation of hard-tissue-forming cells [17]. When calcium hydroxide is applied to the exposed pulp tissue, a layer of dystrophic calcification associated with cellular degeneration may be the surface to which the pulp cells migrate and attach and where they subsequently differentiate into odontoblast-like cells. This process is considered to be mediated by the fibronectin deposited on this layer, which is structurally comparable to the basement membrane [18]. The formation of the tubular dentin-like matrix is preceded by the deposition of fibrodentin, which contains fibronectin [18]. The fibrodentin may be comparable to the mantle dentin observed in developing teeth and has been suggested to play an important role in the terminal differentiation of odontoblasts [13]. Recently, calcium ions released from calcium hydroxide have been shown to stimulate fibronectin gene expression in dental pulp cells [19].

During reparative dentinogenesis after pulp capping, bone sialoprotein (BSP) and osteopontin (OPN) have been detected at the exposure site and in the fibrous matrix (fibrodentin), but not in the tubular dentin-like matrix [20, 21] (Figure 1), while odontoblast-like cells were shown to express dentin sialoprotein [22, 23]. BSP and OPN are suggested to control the mineralization process [24]. These noncollagenous proteins may be associated with the initially formed calcified layer underneath the superficial necrotic zone. In addition, OPN is implicated in diverse biological events, including wound healing [25]. Interestingly, OPN gene expression in human dental pulp cells is enhanced by fibronectin [19]. The colocalization of fibronectin and OPN at the pulp exposure site suggests their role in the migration of progenitors and their differentiation into odontoblast-like cells during reparative dentinogenesis.

Transforming growth factor-*β* (TGF-*β*) and other members of this family of growth factors have been implicated in tooth development and dental tissue repair [26]. TGF-*β* isoforms and their receptors have been identified in odontoblasts of healthy and carious teeth [27, 28], and the TGF-*β* type I receptor was identified in an animal model of pulp capping [20]. TGF- *β*1 has been demonstrated to bind to immobilized fibronectin [29]. Extracellular matrices like proteoglycans have been suggested to bind to many growth factors and modulate their activities [30]. During reparative processes after pulp capping, the fibronectin-rich matrix serves as a reservoir of growth factors as well as a substrate for cell migration and attachment. Such growth factors including TGF-*β* and other inductive molecules expressed in the pulp tissue might be involved in odontoblast-like cell differentiation.

The derivation of the stem/progenitor cells during reparative dentinogenesis is still unclear. Pulp tissue contains a population of cells with stem-cell-like properties. Dental pulp stem cells have been found in human permanent teeth [31, 32] and exfoliated human deciduous teeth [33]. These stem cell populations have been suggested to reside in the microvasculature [34]. Hard tissue formation occurs in the pulp cavity after tooth replantation and transplantation, where dentin- and bone-like tissues are seen [35, 36]. Transplantation of a green fluorescent protein (GFP-) transgenic rat tooth into a wild-type rat socket demonstrated that bone-like tissue was formed by both host and donor cells. On the other hand, all cells lining the dentin-like matrix expressed GFP, suggesting that this matrix was formed by surviving donor odontoblasts and/or pulp cells capable of differentiating into odontoblast-like cells [37]. These results suggest that the dental pulp contains two types of progenitor cells capable of differentiating into either odontoblast- or osteoblast-like cells. This has been confirmed by allogenic tooth transplantation into the sublingual region using lacZ-transgenic ROSA26 mice [38]. Both types of progenitor cells might also be involved in reparative dentinogenesis. It is likely that the superficial layer and the fibrodentin of the newly formed dentin bridge matrix have osteogenic characteristics. The involvement of other cells including inflammatory cells from the bloodstream in pulpal wound healing is not ruled out.

Current studies are focusing on the derivation and phenotypes of the cells involved in nonspecific reparative dentinogenesis and the molecular mechanisms that regulate their cytodifferentiation. Such approaches will ultimately lead to regenerative therapy and tissue engineering of the dentin-pulp complex.

## 3. Cellular Reactions to MTA In Vitro

A number of in vitro studies have been conducted to evaluate the biocompatibility of MTA by measuring various parameters such as proliferation and viability using different types of cells in direct and/or indirect contact with MTA. Overall, the results suggest that the cytotoxicity of set MTA is less than that of traditional materials (reviewed by Camilleri and Pitt Ford [39] and by Roberts et al. [40]). For example, studies using cultured osteoblastic cells have demonstrated that MTA is less toxic than amalgam [41–43], Super EBA [42, 43], and intermediate restorative material (IRM) [41], since cells in contact with MTA showed higher viability and/or proliferative activity. However, MTA in its freshly mixed state shows a higher cytotoxicity [44, 45], which could be due to its high pH [46–49]. 

In vitro experiments have also demonstrated that MTA has the capacity to stimulate cell differentiation/activation, which may contribute to hard tissue matrix formation and/or mineralization. Incubation of gingival and periodontal ligament fibroblasts with MTA causes the induction of osteogenic phenotypes such as alkaline phosphatase, osteonectin, osteopontin, and osteonidgen [43]. MTA also stimulates the production of bone morphogenetic protein (BMP-) 2 and TGF-*β*1 from human gingival fibroblasts [50] and causes the upregulation of type I collagen and osteocalcin mRNA expression in an osteoblast-like cell line (MC3T3-E1) [51]. In a more recent study in which a cementoblast cell line was used, WMTA extracts at lower concentrations induced biomineralization of these cells and caused the upregulation of the mRNA expression of type I collagen and bone sialoprotein [52].

Recent studies using pulp cells have also suggested the capacity of MTA to stimulate matrix formation and mineralization during dentinogenesis. Cultured rat pulp cells stimulated with MTA through transwell inserts showed an increased mineralization and upregulation of BMP-2 mRNA and protein [53]. Thus, BMP-2 may be involved in MTA-induced mineralization. The MTA-induced mineralization together with the alkaline phosphatase activity and dentin sialophosphoprotein (DSPP-) and BSP-expressions of cultured pulp cells was further enhanced when enamel matrix derivative (EMD) was added [54]. Thus, a combination of MTA and EMD may promote the differentiation of pulp cells more rapidly than MTA alone. In addition, rat clonal pulp cells stimulated with MTA upregulate cyclooxygenase-2 and inducible nitric oxide synthase mRNA expression via the nuclear factor kappa B signaling system [55]. Thus, prostaglandins and nitric oxide could be involved in various MTA-induced pulp tissue reactions including inflammation and hard tissue formation.

Overall, in vitro studies indicate that MTA is a biocompatible material and possesses the capacity to stimulate hard tissue-forming cells to induce matrix formation and mineralization. As will be discussed below, a significant part of the bioactivity of MTA in vitro may arise from its reactions with the surrounding environment, for example, the culture media and/or serum [56], which form biocompatible byproducts, including carbonated apatite [57, 58].

## 4. In Vivo Reparative Dentinogenesis by MTA

Subcutaneous and intraosseous implantation studies have consistently shown that MTA elicits less severe tissue reactions compared with those of traditional materials such as amalgam and Super EBA (reviewed by Camilleri and Pitt Ford [39] and Roberts et al. [40]). Moreover, subcutaneous implantation of MTA causes dystrophic calcification in the connective tissue adjacent to this material [59, 60]. 

The ability of MTA to induce reparative dentinogenesis or dentin bridge formation has been consistently demonstrated in animal studies in which direct pulp capping or pulpotomy was performed in mechanically exposed pulps [21, 61–70]. These studies have also shown that MTA causes limited pulp tissue necrosis shortly after its application. Thus, MTA seems less causative compared with calcium hydroxide, which is known to cause the formation of a necrotic layer along the material-pulp interface [1, 9–11]. Compared with calcium hydroxide, MTA induces reparative dentin formation at a greater rate and a superior structural integrity [61, 62]. 

Also, the majority of studies in which MTA capping was carried out in mechanically pulp-exposed healthy human teeth showed that MTA provides higher frequencies of dentin bridge formation, a better quality (thickness, completeness, and/or integrity) dentin bridge, and milder pulp inflammation compared with calcium hydroxide-based materials [23, 71–78]. In one representative study involving 33 healthy third molars [75], direct pulp capping was performed with MTA or a hard-setting calcium hydroxide cement (Dycal) in 20 and 13 teeth, respectively, and histological, ultrastructural, and quantitative analyses were carried out after 1 week to 3 months. The MTA-capped pulps were mostly free from inflammation, and hard tissue bridges of steadily increasing length and thickness were formed. Dycal-capped pulps, however, showed the formation of less consistent barriers, which were frequently accompanied by tunnel defects, and pulp inflammation often persisted, even after 3 months. Moreover, a recent study has reported that MTA pulpotomy in 12 human permanent molars with irreversible pulpitis resulted in complete dentin bridge formation in all cases after 2 months [79].

On the other hand, the cellular and molecular events involved in MTA-induced reparative dentinogenesis have been addressed in a limited number of in vivo studies. In one study, early pulpal cell response after capping with GMTA was examined in mechanically exposed dog pulps [64]. GMTA initially induced the formation of a zone of crystalline structure and an arrangement of pulp cells with the morphological features of increased biosynthetic activity, for example, nuclear and cytoplasmic polarization and developed cytoplasmic organization. Then, the deposition of fibrodentin, followed by reparative dentin formation, which was characterized by the presence of polarized odontoblast-like cells and a tubular dentin-like matrix, was seen. Thus, the stereotypic pulp defense mechanism by which fibrodentin triggers the expression of the odontoblastic potential of pulp cells [10, 13] may be involved in MTA-induced reparative dentinogenesis. In another study, the reparative process of mechanically exposed rat molar pulps capped with WMTA was investigated by immunohistochemistry [21]. The reparative process involved initial deposition of osteopontin in the superficial layer of the pulpal matrix followed by increased cell proliferation and the appearance of nestin-immunoreactive newly differentiated odontoblast-like cells. Thus, the reparative dentinogenesis that occurs following MTA capping is primarily governed by the natural healing process of exposed pulps, which involves the proliferation and migration of progenitors followed by their differentiation into odontoblast-like cells. Osteopontin could play a triggering role in the initiation of this process. The expression of dentin sialoprotein, a noncollagenous protein expressed exclusively by odontoblasts, has been detected in newly differentiated odontoblast-like cells after direct pulp capping of human teeth with MTA [23]. Stronger dentin sialoprotein expression was observed in MTA-capped teeth than in Dycal-capped teeth, suggesting a superior dentinogenic effect of MTA.

Based on the histologic investigations mentioned above, MTA appears superior to calcium hydroxide-based materials with regard to its capacity to stimulate reparative dentinogenesis, as far as mechanically exposed healthy pulps are concerned. MTA and calcium hydroxide share a mechanism of hard tissue formation that can be regarded as the natural healing process of exposed pulps. Thus, MTA should be regarded as “the current gold standard material”, for in vivo pulp capping experiments aimed at investigating the cellular and molecular mechanisms involved in nonspecific reparative dentinogenesis. Based on the in vitro capacity of MTA to stimulate hard tissue-forming cells, however, the possibility that MTA has specific actions for stimulating dentinogenesis cannot be ruled out.

## 5. MTA as a “Calcium Hydroxide-Releasing Material” 

The major component of MTA is essentially a refined preparation of Portland cement, which is a mixture of dicalcium silicate (2CaO · SiO_2_), tricalcium silicate (3CaO · SiO_2_), tricalcium aluminate (3CaO · Al_2_O_3_), gypsum, and tetracalcium aluminoferrite (4CaO · Al_2_O_3_ · Fe_2_O_3_) [5–8]. Gypsum is added for setting retardation. Trace amounts of SiO_2_, CaO, MgO, K_2_SO_4_, and Na_2_SO_4_ are also present [5–7]. The greatest difference in composition between MTA and Portland cement is that MTA contains bismuth oxide as a radiopacifier [5–8]. Moreover, the particles of MTA are more uniform and smaller than those of Portland cement [7]. MTA also contains fewer toxic heavy metals and has a longer working time [80, 81]. Thus, MTA has undergone modification and purification from Portland cement to make it more suitable for clinical use. The major compositional difference between the grey and white versions of MTA is that the levels of chromophores (mainly ferric compounds such as tetracalcium aluminoferrite) are greatly reduced in the white version.

The compressive strength of MTA gradually increases after initial setting and is comparable to those of the Super EBA and IRM cements after 21 days [82]. Thus, MTA possesses sufficient physical strength for endodontic use and is stronger than calcium hydroxide-based materials. Increasing the water-to-powder ratio causes increases in the porosity and solubility of MTA [46]. Too large a condensation pressure causes decreases in surface hardness and compressive strength [83]. 

The setting reaction of MTA is basically similar to that of Portland cement involving the hydration of anhydrous mineral oxide compounds via the dissolution of the compounds followed by the crystallization of hydrates [49, 84, 85]. During the hydration process of calcium silicate components, a portlandite phase, which is mainly composed of calcium hydroxide crystals, is produced together with less basic calcium silicate hydrate (3CaO · 2SiO_2_ · 3H_2_O) as follows [49, 84, 85]:

2(3CaO · SiO_2_) + 6H_2_O

  →3CaO · 2SiO_2_ · 3H_2_O + 3Ca(OH)_2_, 

2(2CaO · SiO_2_) + 4H_2_O

  →3CaO · 2SiO_2_ · 3H_2_O + Ca(OH)_2_. 

MTA is hydrophilic and requires moisture to set, which is a favorable property when there is potential for moisture contamination in the clinical setting; moisture from the surrounding tissue may assist the setting [82, 86]. Blood contamination has little impact on the degree of leakage [87]. One less than ideal property of MTA is that it is a slow-setting material like Portland cement. MTA requires approximately three hours for initial setting [7, 82], and the reaction continues slowly for weeks [82, 88, 89] and probably months. 

Hydrated MTA is alkaline, and its pH rises from 10.5 to 12.5 three hours after mixing [6, 82]. This pH rise is due to the progression of calcium hydroxide formation during the hydration process. When set MTA is immersed in water, it shows solubility of less than 3% of weight loss in 24 hours [46, 81, 82, 90–92], which is lower than that of zinc oxide-eugenol cement [82]. Moreover, Ca ions are continuously released, and the medium maintains a high pH [46–49]. Such dissolution of calcium hydroxide may be a key mechanism behind the biological properties of MTA. 

The dissolution of calcium hydroxide may negatively influence the physical properties of MTA. SEM analysis of water-immersed MTA revealed an increased porosity, which may have been caused by the dissolution of calcium hydroxide and other hydration products [46]. Water immersion of MTA results in the formation of a subsurface layer of low Ca concentration (Ca-leached layer) [93]. A porosity increase and the formation of a Ca-leached layer have also been reported for Portland cement [94, 95] and thus these properties are derived from the parent material. Nevertheless, MTA has advantages over traditional calcium hydroxide preparations in terms of its structural stability and sealing ability, since calcium hydroxide preparations show a high degree of dissolution and lack sealability [96–98]. In addition, Si and Al show an increased concentration within the Ca-leached layer [93], probably resulting from the formation and/or accumulation of insoluble components such as calcium silicate hydrate and ettringite. Thus, the dissolution process may not be one way, but rather involves a “self-reparative” mechanism that compensates for Ca dissolution.

## 6. Interaction of MTA with the Surrounding Environment: A Basis for Its Bioactivity

**Figure 2:**
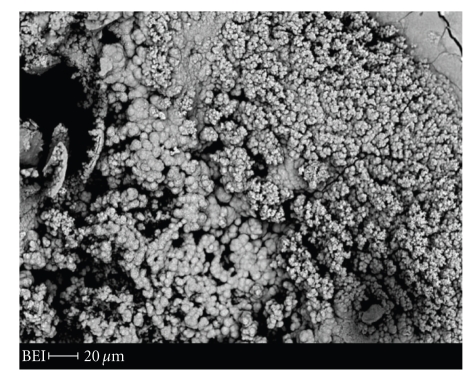
SEM photograph of the surface of white MTA immersed in PBS for 10 days, showing precipitates of various morphologies. Wavelength-dispersive X-ray spectroscopy analysis revealed that the precipitates contained Ca and P as their main elemental components.

MTA and Portland cements are virtually devoid of phosphorus [5–7]. However, when these cements are immersed in phosphate-containing solutions such as phosphate-buffered saline (PBS), they interact with the medium and produce apatite crystals on their surfaces [57, 58, 99–102] (Figure 2). The portlandite phase is not detectable on the upper surface of PBS-immersed Portland cement, but is found only in the interior [102]. This indicates that the calcium ions supplied by portlandite dissolution interact with the phosphate ions in the medium, allowing for the formation of apatite crystals. Such properties of apatite formation are considered to be important for explaining the biocompatibility and/or bioactivity of MTA, since the surface precipitation of biocompatible material(s) may be a basis for the bioactivity of several inorganic biomaterials [103, 104]. The precipitates are also formed at the MTA-dentin interface [57, 58, 99, 105], and thus may play a role in the achievement of a good marginal seal, as will be described below.

Sarkar et al. [99] first reported the formation of white precipitates with a globular ultrastructure on GMTA following immersion in PBS solution. Using X-ray diffraction (XRD) analysis, the authors identified the crystals as hydroxyapatite, although their calcium-to-phosphorus ratios were different from that reported for hydroxyapatite. This finding was confirmed by Bozeman et al. [100], who also used XRD and SEM and analyzed both WMTA and GMTA that had been subjected to PBS immersion. They concluded that the crystal precipitates on both MTA materials were chemically and structurally similar to hydroxyapatite. In addition, the authors also found that GMTA produces twice as many crystals as WMTA, suggesting that the two MTA materials do not possess the same level of bioactivity [100]. On the other hand, Tay et al. [57] analyzed the crystal precipitates on PBS-immersed white Portland cement with SEM, XRD, TEM, and Fourier transformation-infrared spectroscopy. They identified the precipitates as calcium-deficient poorly crystalline carbonated apatite, which had been transformed from an initially formed amorphous calcium phosphate. Most recently, Reyes-Carmona et al. [58] examined the precipitates on various PBS-immersed MTA preparations and Portland cements by means of SEM and XRD and found the presence of amorphous calcium phosphate crystals of different morphologies and Ca/P ratios, which may act as precursors during the formation of carbonated apatite. The formation of crystals mainly composed of Ca and P has also been demonstrated on the pulpal surface of MTA that had been capped on exposed dog pulps in vivo [64].

Taken together, studies have consistently reported that MTA and Portland cement are able to interact with phosphate-containing fluids to form apatite deposits on their surfaces. This appears to be a common characteristic of calcium silicate-containing biomaterials [106, 107]. Since carbonated apatite represents the biological apatite phases found in bone, cementum, and dentin, this apatite layer may play a triggering role in the dentinogenic activity of MTA by supporting new tissue formation and its integration into dentin-like tissue.

## 7. Sealing Ability

A large number of studies have demonstrated that MTA has a better sealing ability than that of traditional materials such as amalgam, glass ionomer cement, and zinc oxide-eugenol cement by means of dye leakage, bacterial leakage, and fluid infiltration tests (reviewed by Roberts et al. [40]). However, the question of why MTA exhibits such a good seal has not yet fully been resolved.

MTA shows slight expansion upon setting [108–110], which may contribute, at least in part, to its good sealing ability. The marginal adaptation of MTA is in general better than that of traditional materials [111–114], although one study [114] reported that marginal adaptation did not correlate with leakage. WMTA exposed to a water-soluble dye before achieving full set showed poorer adaptation and more leakage compared with those of IRM and Super EBA cements [115]. 

GMTA-dentin bond failure usually occurs cohesively within the MTA material [116]. This indicates the presence of certain bonding mechanisms that may contribute to its sealing ability. One explanation for this mechanism is the above-mentioned ability of MTA to spontaneously produce apatite precipitates in the presence of phosphate-containing fluids [57, 58, 99, 100, 102], since materials with an apatite layer are known to form a chemical bond with calcified tissues such as bone [103, 107, 117]. Additionally, the formation of apatite-like materials that fill the MTA-dentin interfacial space has been demonstrated [58, 99]. The apatite was deposited within collagen fibrils, and the interfacial layer composed of apatite was accompanied with tag-like structures that extended into the dentinal tubules [58]. Taken together, the MTA-dentin interfacial layer formation that results from the capacity of MTA to induce spontaneous apatite formation may contribute to minimizing leakage not only by filling the gap along the interface but also via interactions with dentin such as intrafibrillar apatite deposition to promote mineral nucleation on dentin.

## 8. Antimicrobial Activity

Antimicrobial capacity due to high pH is considered one of the advantages of the calcium hydroxide-based materials used for direct pulp capping, since this procedure is often carried out on pulps that have already been bacterially contaminated and/or carry a potential risk of bacterial leakage along the restoration margins [96, 97, 118]. Various MTA preparations show antibacterial [119–125] and antifungal [124, 126–128] activities against different microbial strains. Similar to calcium hydroxide-based materials, the antimicrobial action of MTA is most likely associated with elevated pH resulting from ionization that releases hydroxyl ions. However, this activity of MTA is limited against some facultative bacteria and has no effect on strict anaerobic bacteria [119], and it is also weaker than the actions of zinc oxide-eugenol cements [119, 124]. Sealapex, a calcium hydroxide-based sealer, shows similar to better anti-microbial activity compared with those of various MTA preparations and/or Portland cements [121, 124, 125]. Taken together, the antimicrobial activity of MTA may not be as strong as those of traditional calcium hydroxide-based cements and sealers, although it does contribute to the reduction of bacterial contamination in pulpal wounds. The weaker antimicrobial activity of MTA may be compensated for by its good sealing ability.

## 9. Clinical Performance of MTA as a Pulp Capping Medicament and Pulpotomy Dressing

Two studies have investigated the clinical performance of MTA applied to the pulp capping of cariously exposed permanent teeth and reported success rates of 93% [129] and 98% [130]. In one study [130], 53 teeth with carious exposures diagnosed as reversible pulpitis were capped with MTA. Forty-nine out of 53 teeth were recalled in a mean period of 3.94 years, and 98% of the cases presented a normal radiographic appearance, no symptoms, and a normal response to cold testing. Regarding primary teeth, one study evaluated the clinical outcome of direct pulp capping with either WMTA or Dycal in 25 symmetrical pairs of carious primary molars [131]. None of the teeth exhibited clinical or radiographical failure during the follow-up period of up to 24 months. 

Four studies regarding MTA pulpotomies in cariously exposed permanent teeth have reported high success rates ranging from 93%–100% [132–135]. In one prospective study, the success rates of partial pulpotomies using either GMTA or calcium hydroxide were compared with a mean follow-up period of 34.8 months [135]. Fifty-one teeth in 34 patients were available for recall, and there was no statistically significant difference in the success rate between GMTA and calcium hydroxide (93% and 91%, resp.). In addition, a case report in which partial pulpotomies were conducted on 2 cases of dens evaginatus and histologic examination was conducted after 6 months; complete dentin bridge formation without pulp inflammation was demonstrated [136].

MTA is also used as a pulpotomy dressing for primary teeth and is considered an appropriate alternative to formocresol, since studies comparing MTA and formocresol consistently showed that MTA gave similar to better results both clinically and radiographically [137–143]. 

Overall, the clinical outcome of direct pulp capping and pulpotomy with MTA seems quite favorable, although the number of controled prospective studies is still limited.

## 10. Modification of MTA: A Research Trend

The handling properties of MTA are recognized to be less than ideal, since the working time is limited to a few minutes—even though this slow-setting material requires approximately three hours for initial setting [7, 82]—and the cement mixture is somewhat grainy and sandy. Thus, attempts were made to improve these drawbacks by using additives to accelerate setting. Calcium chloride (2% to 15%) has been widely studied as a setting accelerator: it reduces the setting time [144–147], increases the sealing ability [148], and maintains a high pH [144, 147]. Moreover, the addition of calcium chloride did not affect the formation of dentin bridges following pulpotomies in dog teeth [70], and thus may not deteriorate the biologic properties of MTA. However, calcium chloride reduces the compressive strength of set MTA [144]. One study recommended an admix of 1% methylcellulose and 2% calcium chloride because it improved the handling properties of MTA without reducing its compressive strength [145]. Na_2_HPO_4_ also reduces the setting time [149] while maintaining biocompatibility in vitro [150]. A recent report has documented that the addition of Na_2_HPO_4_ to WMTA creates a more biocompatible material, as demonstrated by subcutaneous implantation [60]. However, MTA with the addition of resinous components to allow light curing did not stimulate mineralization when implanted into rat connective tissues [151]. 

Attempts have also been made to improve the working (and physical) properties of MTA by developing new calcium silicate-based materials [108, 152–157]. Among these, NEC (new endodontic cement) is a novel endodontic material consisting of different calcium compounds (i.e., calcium oxide, calcium phosphate, calcium carbonate, calcium silicate, calcium sulfate, calcium hydroxide, and calcium chloride) [155–157]. NEC is reported to show a shorter setting time [155], better handling properties, and a similar sealing ability [157] compared with those of MTA. When applied to exposed dog dental pulps, both NEC and MTA show favorable responses characterized by the formation of dentin bridges, while Dycal showed inferior responses accompanied by incomplete dentin bridge formation and pulp inflammation [156].

## 11. Conclusions

The available literature suggests that MTA is more efficient at inducing reparative dentinogenesis in vivo compared with calcium hydroxide-based materials. However, MTA and calcium hydroxide share several biological properties that contribute to the induction of reparative dentinogenesis, mostly due to the fact that set MTA acts as a “calcium hydroxide-releasing material.” Thus, the dentinogenic mechanism of MTA may be attributable to the natural wound healing process of exposed pulps, which is considered to be the mechanism involved in calcium hydroxide-induced reparative dentinogenesis. Nevertheless, in vitro studies suggest the presence of dentinogenic mechanisms specific to MTA, since MTA can stimulate hard tissue-forming cells to induce matrix formation and mineralization. MTA has several beneficial physical properties over calcium hydroxide, including a good sealing ability, a lower degree of dissolution, and a higher structural stability. MTA also has the ability to interact with phosphate-containing fluids to spontaneously form apatite precipitates, which not only explains its biocompatibility and bioactivity but may also contribute to its sealing ability. Thus, the capacity of MTA to induce hard tissue repair of exposed pulps may depend heavily on its ability to create a local environment in which the inherent wound healing capacity of the pulp is not deteriorated. 

The clinical outcome of direct pulp capping with MTA seems quite favorable, although the number of controled prospective studies is still limited. Attempts are being conducted to improve the working properties of MTA via the addition of setting accelerators and the development of new calcium silicate-based materials.
